# Supplementary material for: Saccharomyces boulardii (CNCM I-745) improves intestinal damage in sepsis by remodeling intestinal flora structure
Source: Front Cell Infect Microbiol. 2025 Jul 31;15:1602792. doi: 10.3389/fcimb.2025.1602792 (PMC12350329; doi:10.3389/fcimb.2025.1602792)
Supplement: Supplementary file 1 [file DataSheet1.zip › summary/summary/8_Advanced_analyse/2_PICRUSt2_result/KEGG/Group.DW4_vs_BW4/PICRUSt2_KEGG_level3_Group.DW4_vs_BW4_diff.pdf]

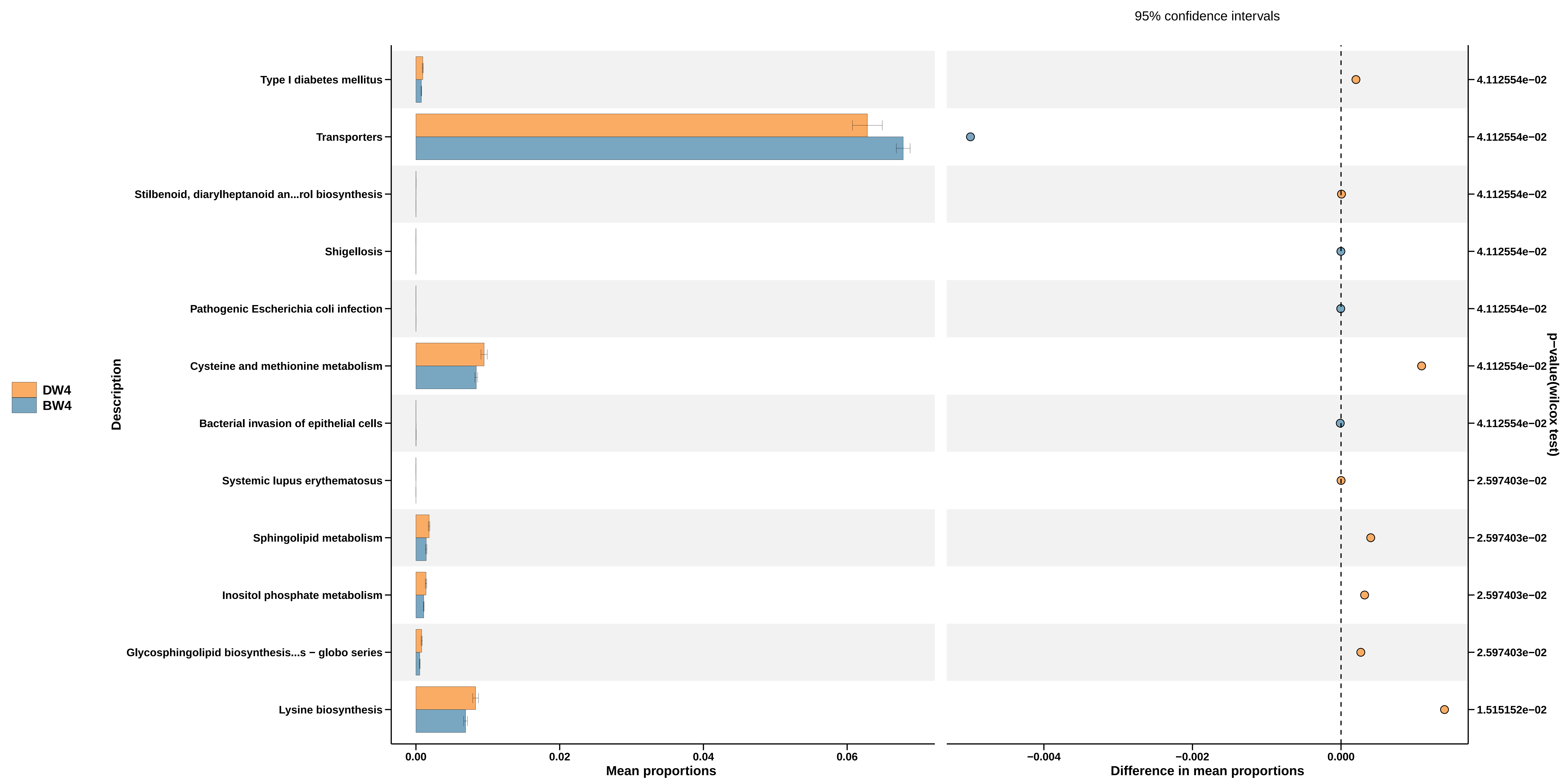
95% confidence intervals
DW4
BW4
Description
Type I diabetes mellitus
Transporters
Stilbenoid, diarylheptanoid an...rol biosynthesis
Shigellosis
Pathogenic Escherichia coli infection
Cysteine and methionine metabolism
Bacterial invasion of epithelial cells
Systemic lupus erythematosus
Sphingolipid metabolism
Inositol phosphate metabolism
Glycosphingolipid biosynthesis...s − globo series
Lysine biosynthesis
0.00
0.02
0.04
0.06
Mean proportions
−0.004
−0.002
0.000
Difference in mean proportions
4.112554e−02
4.112554e−02
4.112554e−02
4.112554e−02
4.112554e−02
4.112554e−02
4.112554e−02
2.597403e−02
2.597403e−02
2.597403e−02
2.597403e−02
1.515152e−02
p−value(wilcox test)
